# The relationship between spiritual, religious and personal beliefs and disordered eating psychopathology

**DOI:** 10.1186/2050-2974-3-S1-P3

**Published:** 2015-11-23

**Authors:** Daniel Akrawi, Shantiban Shanmugam, Michael Lee, Rohit Ghosh, Stephen Touyz, Roger Bartrop

**Affiliations:** 1 University of Western Sydney, Sydney, Australia; 2 University of Sydney, Sydney, Australia

## Objectives

With high rates of eating disorders and disordered eating amongst female university students, new paradigms in prevention are needed. Thus, this study aims to explore the relationships between disordered eating psychopathology (DEP) and spiritual, religious and personal belief (SRPB) facets amongst female university students in Western Sydney.

## Design

A cross-sectional study was conducted surveying female university students attending the University of Western Sydney (UWS). The Eating Disorder Inventory-3 (Drive for Thinness, Bulimia, Body Dissatisfaction scales) and the World Health Organisation Quality of Life - Spiritual, Religious and Personal Beliefs BREF questionnaires were used.

## Participants

A total of 616 female participants from UWS were recruited through university emails, social media and community engagement across all university campuses. The participants had a mean age of 21.45 years and a mean BMI of 23.8.

## Results

Students who described themselves as being more religious had lower levels of body dissatisfaction. Furthermore, all 9 SRPB facets were correlated with lower levels of DEP. However, wholeness & integration, and to a lesser extent inner peace derived from strong SRPBs were found to best predict lower levels of DEP.

## Conclusions

Significant associations were found between SRPBs and lower levels of DEP. These results can be used to inform health professionals, as well as religious and spiritual leaders in understanding, addressing and preventing disordered eating in Western Sydney.

